# (1-Oxo-2,6,7-trioxa-1-phosphabicyclo­[2.2.2]octan-4-yl)methyl 4-methyl­benzene­sulfonate

**DOI:** 10.1107/S1600536812016674

**Published:** 2012-04-21

**Authors:** Xu-Feng Hou, Jin-Long Yan

**Affiliations:** a School of Chemistry and Chemical Engineering, Xuchang University, Xuchang, Henan Province, 461000, People’s Republic of China; b Department of Biology and Chemistry, Jiaozuo Teachers’ College, Jiaozuo, Henan Province, 454000, People’s Republic of China

## Abstract

In the title compound, C_12_H_15_O_7_PS, the P atom has a distorted tetra­hedral environment. The P—O—C—C torsion angles deviate significantly from zero [average = 12.0 (3)°], indicating that the bicyclic OP(OCH_2_)_3_C cage is strained. In the crystal, weak C—H⋯O inter­actions consolidate the packing.

## Related literature

For related structures, see: Miu *et al.* (1991 ▶); Sheng & He (2006 ▶); Guo & Zang (2007 ▶). For applications of caged bicyclic phosphates and *p*-toluene­sulfonates, see: Li *et al.* (2000 ▶); Yachi *et al.* (1989 ▶); Spungin *et al.* (1992 ▶).
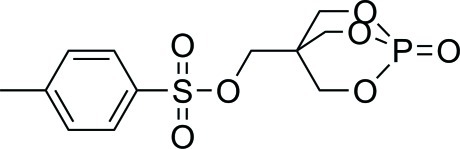


## Experimental

### 

#### Crystal data

C_12_H_15_O_7_PS
*M* *_r_* = 334.27
Monoclinic, 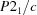

*a* = 5.8884 (17) Å
*b* = 19.440 (5) Å
*c* = 12.469 (4) Å
β = 100.614 (4)°
*V* = 1402.8 (7) Å^3^
*Z* = 4
Mo *K*α radiation
μ = 0.38 mm^−1^
*T* = 113 K
0.20 × 0.18 × 0.12 mm

#### Data collection

Rigaku Saturn CCD area-detector diffractometer
Absorption correction: multi-scan (*CrystalClear*; Rigaku/MSC, 2005 ▶) *T*_min_ = 0.929, *T*_max_ = 0.956
17318 measured reflections
3353 independent reflections
2808 reflections with *I* > 2σ(*I*)
*R*_int_ = 0.042

#### Refinement

*R*[*F* ^2^ > 2σ(*F* ^2^)] = 0.034
*wR*(*F* ^2^) = 0.095
*S* = 1.03
3353 reflections
191 parameters
H-atom parameters constrained
Δρ_max_ = 0.41 e Å^−3^
Δρ_min_ = −0.36 e Å^−3^

### 

Data collection: *CrystalClear* (Rigaku/MSC, 2005 ▶); cell refinement: *CrystalClear*; data reduction: *CrystalClear*; program(s) used to solve structure: *SHELXS97* (Sheldrick, 2008 ▶); program(s) used to refine structure: *SHELXL97* (Sheldrick, 2008 ▶); molecular graphics: *CrystalStructure* (Rigaku/MSC, 2005 ▶); software used to prepare material for publication: *CrystalStructure*.

## Supplementary Material

Crystal structure: contains datablock(s) global, I. DOI: 10.1107/S1600536812016674/cv5286sup1.cif

Structure factors: contains datablock(s) I. DOI: 10.1107/S1600536812016674/cv5286Isup2.hkl

Supplementary material file. DOI: 10.1107/S1600536812016674/cv5286Isup3.cml

Additional supplementary materials:  crystallographic information; 3D view; checkCIF report

## Figures and Tables

**Table 1:** Hydrogen-bond geometry (Å, °)

*D*—H⋯*A*	*D*—H	H⋯*A*	*D*⋯*A*	*D*—H⋯*A*
C7—H7*A*⋯O5^i^	0.98	2.59	3.288 (2)	128
C8—H8*A*⋯O1^ii^	0.99	2.38	3.180 (2)	138
C10—H10*A*⋯O7^iii^	0.99	2.27	3.211 (2)	158
C10—H10*B*⋯O2^iv^	0.99	2.44	3.348 (2)	152

Symmetry codes: (i) 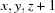; (ii) 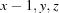; (iii) 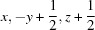; (iv) 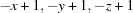.

**Fig. 1.**
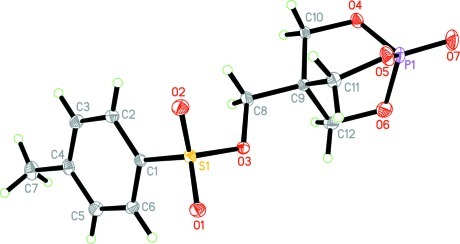
The molecular structure of (I) showing the atomic numbering and 30% probability displacement ellipsoids.

## supplementary crystallographic information

### Comment

Caged bicyclic phosphates are widely used as flame retardants or pesticides (Li *et al.*, 2000). They have been studied in the context of hydrogen-bond patterns (Guo & Zang, 2007). *p*-Toluenesulfonates are also used in monitoring the merging of lipids (Yachi *et al.*, 1989), studying membrane fusion during acrosome reaction (Spungin *et al.*, 1992). We report here the crystal structure of the title compound (I).

In (I) (Fig. 1), all bond lengths and angles are normal and correspond to those observed in the related compounds (Miu *et al.*, 1991; Sheng & He, 2006; Guo & Zang, 2007). The P—O—C—C torsion angles deviate significantly from zero [averaged -12.0 (3)°] indicating that bicyclic OP(OCH_2_)_3_C cage is strained. In the crystal, weak C—H···O interactions (Table 1) consolidate the packing.

### Experimental

The title compound was obtained in the reaction of 4-(hydroxymethyl)-2,6,7-trioxa-1-phosphabicyclo[2.2.2]octane 1-oxide (5 mmol) and 4-toluenesulfonyl chloride(4.7 mmol) in refluxing acetonitrile (50 ml). The solvent was evaporated *in vacuo*. The title compound was recrystallized from ethanol and single crystals of (I) were obtained by slow evaporation.

### Refinement

All H atoms were placed in calculated positions, with C—H = 0.95 - 0.99 Å, and included in the final cycles of refinement using a riding model, with *U*_iso_(H) = 1.2-1.5 *U*_eq_(C).

### Crystal data

**Table:**

C_12_H_15_O_7_PS	*F*(000) = 696
*M**_r_* = 334.27	*D*_x_ = 1.583 Mg m^−^^3^
Monoclinic, *P*2_1_/*c*	Mo *K*α radiation, λ = 0.71073 Å
*a* = 5.8884 (17) Å	Cell parameters from 4690 reflections
*b* = 19.440 (5) Å	θ = 1.0–27.9°
*c* = 12.469 (4) Å	µ = 0.38 mm^−^^1^
β = 100.614 (4)°	*T* = 113 K
*V* = 1402.8 (7) Å^3^	Prism, colorless
*Z* = 4	0.20 × 0.18 × 0.12 mm

### Data collection

**Table:**

Rigaku Saturn CCD area-detector diffractometer	3353 independent reflections
Radiation source: rotating anode	2808 reflections with *I* > 2σ(*I*)
Multilayer monochromator	*R*_int_ = 0.042
Detector resolution: 14.63 pixels mm^-1^	θ_max_ = 27.9°, θ_min_ = 2.0°
ω and φ scans	*h* = −7→7
Absorption correction: multi-scan (*CrystalClear*; Rigaku/MSC, 2005)	*k* = −25→25
*T*_min_ = 0.929, *T*_max_ = 0.956	*l* = −16→16
17318 measured reflections	

### Refinement

**Table:**

Refinement on *F*^2^	Primary atom site location: structure-invariant direct methods
Least-squares matrix: full	Secondary atom site location: difference Fourier map
*R*[*F*^2^ > 2σ(*F*^2^)] = 0.034	Hydrogen site location: inferred from neighbouring sites
*wR*(*F*^2^) = 0.095	H-atom parameters constrained
*S* = 1.03	*w* = 1/[σ^2^(*F*_o_^2^) + (0.056*P*)^2^ + 0.0836*P*] where *P* = (*F*_o_^2^ + 2*F*_c_^2^)/3
3353 reflections	(Δ/σ)_max_ = 0.001
191 parameters	Δρ_max_ = 0.41 e Å^−^^3^
0 restraints	Δρ_min_ = −0.36 e Å^−^^3^

### Special details

**Table:**

Geometry. All e.s.d.'s (except the e.s.d. in the dihedral angle between two l.s. planes) are estimated using the full covariance matrix. The cell e.s.d.'s are taken into account individually in the estimation of e.s.d.'s in distances, angles and torsion angles; correlations between e.s.d.'s in cell parameters are only used when they are defined by crystal symmetry. An approximate (isotropic) treatment of cell e.s.d.'s is used for estimating e.s.d.'s involving l.s. planes.
Refinement. Refinement of *F*^2^ against ALL reflections. The weighted *R*-factor *wR* and goodness of fit *S* are based on *F*^2^, conventional *R*-factors *R* are based on *F*, with *F* set to zero for negative *F*^2^. The threshold expression of *F*^2^ > σ(*F*^2^) is used only for calculating *R*-factors(gt) *etc*. and is not relevant to the choice of reflections for refinement. *R*-factors based on *F*^2^ are statistically about twice as large as those based on *F*, and *R*- factors based on ALL data will be even larger.

### Fractional atomic coordinates and isotropic or equivalent isotropic displacement parameters (Å^2^)

**Table:**

	*x*	*y*	*z*	*U*_iso_*/*U*_eq_	
S1	0.99063 (6)	0.451050 (18)	0.67038 (3)	0.01842 (11)	
P1	0.39289 (8)	0.28254 (2)	0.29030 (3)	0.02821 (13)	
O1	1.22894 (17)	0.45267 (5)	0.66257 (9)	0.0236 (2)	
O2	0.84692 (19)	0.50895 (5)	0.63399 (9)	0.0278 (3)	
O3	0.89302 (16)	0.38603 (5)	0.60079 (8)	0.0214 (2)	
O4	0.20805 (18)	0.29396 (5)	0.36462 (8)	0.0237 (3)	
O5	0.50318 (19)	0.35557 (6)	0.28262 (8)	0.0312 (3)	
O6	0.5883 (2)	0.23944 (6)	0.36336 (10)	0.0380 (3)	
O7	0.3039 (2)	0.25143 (8)	0.18528 (10)	0.0495 (4)	
C1	0.9609 (2)	0.43084 (7)	0.80401 (11)	0.0181 (3)	
C2	0.7696 (3)	0.45395 (8)	0.84399 (13)	0.0244 (3)	
H2	0.6535	0.4805	0.7994	0.029*	
C3	0.7513 (3)	0.43764 (8)	0.95002 (13)	0.0263 (3)	
H3	0.6201	0.4530	0.9776	0.032*	
C4	0.9193 (3)	0.39949 (8)	1.01712 (12)	0.0237 (3)	
C5	1.1086 (3)	0.37578 (8)	0.97464 (13)	0.0261 (3)	
H5	1.2237	0.3488	1.0191	0.031*	
C6	1.1302 (3)	0.39112 (8)	0.86861 (12)	0.0229 (3)	
H6	1.2591	0.3748	0.8401	0.028*	
C7	0.8998 (3)	0.38445 (10)	1.13373 (13)	0.0340 (4)	
H7A	0.7525	0.4022	1.1480	0.051*	
H7B	1.0276	0.4067	1.1830	0.051*	
H7C	0.9068	0.3346	1.1460	0.051*	
C8	0.6458 (2)	0.37224 (9)	0.58979 (12)	0.0258 (4)	
H8A	0.5611	0.4157	0.5953	0.031*	
H8B	0.6176	0.3411	0.6488	0.031*	
C9	0.5622 (2)	0.33904 (7)	0.47941 (11)	0.0170 (3)	
C10	0.2981 (2)	0.33360 (8)	0.46400 (11)	0.0186 (3)	
H10A	0.2546	0.3106	0.5281	0.022*	
H10B	0.2294	0.3802	0.4580	0.022*	
C11	0.6235 (3)	0.38303 (8)	0.38722 (12)	0.0224 (3)	
H11A	0.5763	0.4313	0.3957	0.027*	
H11B	0.7925	0.3821	0.3898	0.027*	
C12	0.6632 (3)	0.26691 (8)	0.47338 (13)	0.0255 (3)	
H12A	0.8340	0.2690	0.4908	0.031*	
H12B	0.6096	0.2364	0.5273	0.031*	

### Atomic displacement parameters (Å^2^)

**Table:**

	*U*^11^	*U*^22^	*U*^33^	*U*^12^	*U*^13^	*U*^23^
S1	0.0172 (2)	0.0208 (2)	0.01733 (19)	0.00029 (14)	0.00335 (14)	0.00003 (13)
P1	0.0281 (2)	0.0345 (3)	0.0219 (2)	0.00056 (18)	0.00434 (18)	−0.01078 (18)
O1	0.0179 (6)	0.0302 (6)	0.0238 (6)	−0.0038 (4)	0.0065 (4)	0.0001 (4)
O2	0.0312 (6)	0.0268 (6)	0.0249 (6)	0.0084 (5)	0.0036 (5)	0.0044 (5)
O3	0.0144 (5)	0.0287 (6)	0.0210 (5)	−0.0011 (4)	0.0032 (4)	−0.0062 (4)
O4	0.0219 (6)	0.0286 (6)	0.0199 (5)	−0.0056 (4)	0.0022 (4)	−0.0036 (4)
O5	0.0316 (6)	0.0469 (8)	0.0159 (5)	−0.0085 (5)	0.0063 (5)	0.0038 (5)
O6	0.0359 (7)	0.0306 (7)	0.0440 (7)	0.0096 (5)	−0.0019 (6)	−0.0168 (6)
O7	0.0439 (8)	0.0701 (10)	0.0329 (7)	−0.0012 (7)	0.0026 (6)	−0.0301 (7)
C1	0.0176 (7)	0.0202 (7)	0.0164 (7)	−0.0015 (6)	0.0029 (6)	−0.0019 (6)
C2	0.0186 (8)	0.0310 (9)	0.0234 (8)	0.0028 (6)	0.0032 (6)	−0.0026 (6)
C3	0.0214 (8)	0.0332 (9)	0.0267 (8)	−0.0017 (6)	0.0105 (7)	−0.0058 (7)
C4	0.0277 (8)	0.0230 (8)	0.0212 (8)	−0.0085 (6)	0.0064 (6)	−0.0037 (6)
C5	0.0294 (8)	0.0260 (8)	0.0225 (8)	0.0035 (7)	0.0040 (6)	0.0037 (6)
C6	0.0207 (7)	0.0259 (8)	0.0227 (8)	0.0041 (6)	0.0053 (6)	−0.0003 (6)
C7	0.0434 (11)	0.0379 (10)	0.0231 (8)	−0.0095 (8)	0.0128 (8)	−0.0014 (7)
C8	0.0160 (7)	0.0417 (10)	0.0204 (8)	−0.0065 (6)	0.0051 (6)	−0.0080 (7)
C9	0.0167 (7)	0.0192 (7)	0.0158 (7)	−0.0011 (5)	0.0044 (5)	0.0004 (5)
C10	0.0179 (7)	0.0240 (7)	0.0143 (7)	−0.0017 (6)	0.0036 (5)	−0.0009 (6)
C11	0.0227 (8)	0.0244 (8)	0.0212 (7)	−0.0049 (6)	0.0072 (6)	0.0018 (6)
C12	0.0226 (8)	0.0227 (8)	0.0297 (8)	0.0029 (6)	0.0006 (7)	0.0013 (6)

### Geometric parameters (Å, º)

**Table:**

S1—O1	1.4248 (11)	C4—C7	1.508 (2)
S1—O2	1.4309 (11)	C5—C6	1.384 (2)
S1—O3	1.5803 (11)	C5—H5	0.9500
S1—C1	1.7518 (15)	C6—H6	0.9500
P1—O7	1.4499 (13)	C7—H7A	0.9800
P1—O4	1.5698 (11)	C7—H7B	0.9800
P1—O5	1.5714 (13)	C7—H7C	0.9800
P1—O6	1.5717 (13)	C8—C9	1.5177 (19)
O3—C8	1.4617 (17)	C8—H8A	0.9900
O4—C10	1.4724 (16)	C8—H8B	0.9900
O5—C11	1.4654 (18)	C9—C11	1.528 (2)
O6—C12	1.4623 (19)	C9—C12	1.530 (2)
C1—C2	1.387 (2)	C9—C10	1.5352 (19)
C1—C6	1.394 (2)	C10—H10A	0.9900
C2—C3	1.383 (2)	C10—H10B	0.9900
C2—H2	0.9500	C11—H11A	0.9900
C3—C4	1.387 (2)	C11—H11B	0.9900
C3—H3	0.9500	C12—H12A	0.9900
C4—C5	1.397 (2)	C12—H12B	0.9900
			
O1—S1—O2	119.56 (7)	C4—C7—H7B	109.5
O1—S1—O3	104.08 (6)	H7A—C7—H7B	109.5
O2—S1—O3	108.73 (7)	C4—C7—H7C	109.5
O1—S1—C1	110.02 (7)	H7A—C7—H7C	109.5
O2—S1—C1	108.73 (7)	H7B—C7—H7C	109.5
O3—S1—C1	104.64 (7)	O3—C8—C9	108.30 (11)
O7—P1—O4	114.69 (7)	O3—C8—H8A	110.0
O7—P1—O5	113.99 (8)	C9—C8—H8A	110.0
O4—P1—O5	104.33 (6)	O3—C8—H8B	110.0
O7—P1—O6	113.91 (8)	C9—C8—H8B	110.0
O4—P1—O6	104.27 (7)	H8A—C8—H8B	108.4
O5—P1—O6	104.49 (7)	C8—C9—C11	111.23 (12)
C8—O3—S1	116.97 (9)	C8—C9—C12	111.73 (12)
C10—O4—P1	112.92 (8)	C11—C9—C12	109.15 (12)
C11—O5—P1	114.35 (9)	C8—C9—C10	107.13 (11)
C12—O6—P1	114.27 (9)	C11—C9—C10	108.58 (12)
C2—C1—C6	120.88 (14)	C12—C9—C10	108.94 (12)
C2—C1—S1	119.94 (11)	O4—C10—C9	109.93 (11)
C6—C1—S1	119.18 (11)	O4—C10—H10A	109.7
C3—C2—C1	118.73 (14)	C9—C10—H10A	109.7
C3—C2—H2	120.6	O4—C10—H10B	109.7
C1—C2—H2	120.6	C9—C10—H10B	109.7
C2—C3—C4	121.78 (15)	H10A—C10—H10B	108.2
C2—C3—H3	119.1	O5—C11—C9	108.85 (12)
C4—C3—H3	119.1	O5—C11—H11A	109.9
C3—C4—C5	118.54 (14)	C9—C11—H11A	109.9
C3—C4—C7	120.85 (15)	O5—C11—H11B	109.9
C5—C4—C7	120.60 (15)	C9—C11—H11B	109.9
C6—C5—C4	120.75 (15)	H11A—C11—H11B	108.3
C6—C5—H5	119.6	O6—C12—C9	109.14 (12)
C4—C5—H5	119.6	O6—C12—H12A	109.9
C5—C6—C1	119.30 (14)	C9—C12—H12A	109.9
C5—C6—H6	120.4	O6—C12—H12B	109.9
C1—C6—H6	120.4	C9—C12—H12B	109.9
C4—C7—H7A	109.5	H12A—C12—H12B	108.3
			
O1—S1—O3—C8	−177.65 (10)	C2—C3—C4—C7	177.82 (15)
O2—S1—O3—C8	−49.18 (12)	C3—C4—C5—C6	1.2 (2)
C1—S1—O3—C8	66.86 (11)	C7—C4—C5—C6	−178.19 (15)
O7—P1—O4—C10	−172.57 (10)	C4—C5—C6—C1	0.1 (2)
O5—P1—O4—C10	−47.17 (10)	C2—C1—C6—C5	−1.2 (2)
O6—P1—O4—C10	62.18 (11)	S1—C1—C6—C5	179.22 (12)
O7—P1—O5—C11	−171.46 (10)	S1—O3—C8—C9	148.31 (10)
O4—P1—O5—C11	62.71 (11)	O3—C8—C9—C11	−54.43 (17)
O6—P1—O5—C11	−46.49 (11)	O3—C8—C9—C12	67.82 (16)
O7—P1—O6—C12	−174.29 (11)	O3—C8—C9—C10	−172.94 (12)
O4—P1—O6—C12	−48.55 (12)	P1—O4—C10—C9	−12.41 (14)
O5—P1—O6—C12	60.69 (12)	C8—C9—C10—O4	−172.94 (12)
O1—S1—C1—C2	152.36 (12)	C11—C9—C10—O4	66.84 (15)
O2—S1—C1—C2	19.67 (15)	C12—C9—C10—O4	−51.92 (15)
O3—S1—C1—C2	−96.37 (13)	P1—O5—C11—C9	−12.39 (15)
O1—S1—C1—C6	−28.07 (14)	C8—C9—C11—O5	−169.66 (12)
O2—S1—C1—C6	−160.76 (12)	C12—C9—C11—O5	66.61 (15)
O3—S1—C1—C6	83.21 (13)	C10—C9—C11—O5	−52.01 (15)
C6—C1—C2—C3	0.9 (2)	P1—O6—C12—C9	−11.31 (16)
S1—C1—C2—C3	−179.56 (12)	C8—C9—C12—O6	−176.46 (12)
C1—C2—C3—C4	0.6 (2)	C11—C9—C12—O6	−53.03 (16)
C2—C3—C4—C5	−1.6 (2)	C10—C9—C12—O6	65.37 (15)

### Hydrogen-bond geometry (Å, º)

**Table:**

*D*—H···*A*	*D*—H	H···*A*	*D*···*A*	*D*—H···*A*
C7—H7*A*···O5^i^	0.98	2.59	3.288 (2)	128
C8—H8*A*···O1^ii^	0.99	2.38	3.180 (2)	138
C10—H10*A*···O7^iii^	0.99	2.27	3.211 (2)	158
C10—H10*B*···O2^iv^	0.99	2.44	3.348 (2)	152

Symmetry codes: (i) *x*, *y*, *z*+1; (ii) *x*−1, *y*, *z*; (iii) *x*, −*y*+1/2, *z*+1/2; (iv) −*x*+1, −*y*+1, −*z*+1.
